# The effect of occupational stress on depression and insomnia: a cross-sectional study among employees in a Ghanaian telecommunication company

**DOI:** 10.1186/s12889-020-08744-z

**Published:** 2020-07-01

**Authors:** Emma Edinam Kploanyi, Duah Dwomoh, Mawuli Dzodzomenyo

**Affiliations:** 1 Department of Biological, Environmental and Occupational Health Sciences, School of Public Health, University of Ghana, Accra, Ghana; 2 Department of Biostatistics, School of Public Health, University of Ghana, Accra, Ghana

**Keywords:** Psychiatric conditions, Psychosocial stressors, Workload, Workplace, Job stress

## Abstract

**Background:**

Depression and insomnia are major psychiatric conditions predicted by occupational stress. However, the influence of occupational stress on these two conditions is under-explored in telecommunication companies, especially in Africa. This research was conducted to assess occupational stress in a Ghanaian telecommunication company and its effect on depression and insomnia.

**Methods:**

An analytical cross-sectional study was conducted among employees at a telecommunication company in Accra. Structured self-administered questionnaires were used in collecting data from 235 respondents using simple random sampling. The Chi-square test of independence and Wilcoxon Rank-Sum test were employed to assess the significance of associations with subsequent sensitivity analysis using Multiple logistic, Poisson and Probit regression models. Occupational stress was matched on four variables: age of the workers, marital status, responsibility for dependents and work experience, to improve on the estimation of its impact on symptomatic depression and insomnia using the coarsened exact matching procedure.

**Results:**

More males (52.8%) than females participated in this study. The age range for study participants was 20–49 years with a mean of 30.8 ± 6.9 years. The prevalence of excessive occupational stress reported by the employees was 32.8% (95% CI = 26.7–38.8). More than half of respondents (51%) reported depressive symptoms in the past week and only a few (6%) reported being diagnosed with insomnia in the past year. Age, responsibility for dependents and work experience were the only background characteristics that were significantly associated with excessive occupational stress. After controlling for background characteristics, the estimated risk of reporting symptoms of depression among employees who reported excessive stress from work was only 5% higher [ARR; 95% CI = 1.05 (0.94–1.17)] whereas it was 2.58 times the risk of reporting insomnia [ARR; 95% CI = 2.58(0.83–8.00)] compared to those who did not report excessive stress from their jobs. The relative risk reduced to 2.46[ARR; 95% CI = 2.46(0.77–7.87)] and 1.03[ARR; 95% CI = 1.03(0.91–1.17)] for insomnia and depression respectively after employing Poisson regression with CEM.

**Conclusion:**

The study found a higher risk of insomnia among employees who reported excessive occupational stress compared to those who did not. However, this study did not find a statistically significant relationship between depression and occupational stress.

## Background

There are variations in how individuals express vulnerability to stress based on modifications in neural mechanisms fashioned to properly tune and terminate stress responses [1]. Coping strategies adopted toward stress are also just as much different due to individual characteristics. These characteristics including age, gross monthly salary, work experience, educational qualification, marital status, and gender have been reported as contributing to differences in the level of occupational stress [2–4].

Occupational strain resulting from exposure to stressors at the workplace can affect an employee’s mental and physical health. Depression, the leading cause of disability and the main indication of psychiatric conditions, as well as insomnia, are major psychological disorders reported to be significantly associated with occupational stress [5–7]. High levels of occupational stress do not only predict insomnia but also its recurrence [8]. Fatigue after work and high levels of work rumination could mediate this relationship between occupational stress and insomnia [9]. Moreover, occupational stress has been implicated in the early onset of insomnia, which is before 30 years of age [10]. In a recent longitudinal study [11], it was found that sleeping problems mediate the relationship between stress and metabolic syndrome; insomnia caused by occupational stress showed a highly significant association with the latter.

Workload is known to be a major stressor in the fast-growing and contemporary service industry globally and the telecommunication companies (telcos) are no exceptions [12]. Moreover, a number of suicides informed by occupational stress in some telcos [13] indicate its influence on the psychological wellbeing of employees. Despite this, there is very little information on the extent of stress workload exerts on the workforce in telcos and also its psychosocial consequences on their workers in Africa. This study, therefore, aims to assess occupational stress in a Ghanaian telco and quantify its possible effect on two psychiatric conditions, depression and insomnia among the workers. The findings are intended to serve as evidence for employers to assess psychosocial hazards at their workplaces and effectively manage them so as to protect workforce health with the ultimate goal of improving productivity.

## Methods

### Selection and description of participants

Ethical approval (GHS-ERC Number: 046/01/18) was obtained from the Ghana Health Service Ethical Review Committee of the Research and Development Division, Accra, before conducting this analytical cross-sectional study. The study population comprised of employees, both permanent and contract staff, working at the Head Office of a Ghanaian telco in Accra. Study participants were selected by simple random sampling proportionate to the size of their departments. By this, all employees in this organization had equal opportunities to be part of the study and the diversity in their job descriptions were also catered for.

### Data collection tool and technique

Data were collected on the variables of interest using a structured self-administered questionnaire constructed in English and adapted from the National Institute for Occupational Safety and Health (NIOSH) Generic Job Stress Questionnaire (GJSQ) [14]. This tool has shown high validity and reliability: (0.65–0.90) [15] and (0.68–0.95) [16, 17]. The questionnaire was divided into three sections; the first section gathered data on respondents’ sociodemographic and job characteristics. Occupational stress was measured using a composite scale under four domains of workplace psychosocial stressors in section two: quantitative workload (4 items; 5-point Likert scale), responsibility for others (4 items; 5-point Likert scale), role conflict (4 items;7-point Likert scale) and role ambiguity (5 items; 7-point Likert scale). In section three, the Center for Epidemiologic Studies Depression Scale (CES-D) as employed in the GJSQ was used to measure depression (reported symptoms of depression for the past 1 week) using a composite scale (14 items; 4-point Likert scale) whereas insomnia (reported clinical diagnosis of insomnia in the past 1 year) was measured using a single item with a binary response (1 = Yes, 0 = No). Also, two items in this section obtained respondent information on two lifestyle patterns: smoking and alcohol intake. Scoring keys provided by NIOSH GJSQ were used to convert responses into quantitative measurements for analysis. The maximum period of time estimated for filling out a questionnaire was 10 min. Data collection was carried out for 5 weeks concurrently with data entry and cleaning.

### Statistical analysis

Respondents’ data from completed questionnaires were entered manually into an excel form (Excel version 2016). A coding frame containing the scores corresponding to the responses on the questionnaires served as a guide for data entry to aid ‘translate’ the survey data into a workable dataset.

Descriptive (mean, median) and inferential statistics (hypothesis testing) were obtained from data gathered using the STATA version 15 software. The various ranges of scores for composite variables were as follows: workload and responsibility for others (0–16); role ambiguity (0–30); role conflict (0–24); overall occupational stress (0–86); depression (0–42) and work productivity (0–60). Higher scores implied higher reported occupational stress and depression respectively. Total scores for each composite variable were converted into percentages before categorizing them. Overall occupational stress and the stress from the four psychosocial stressors were first categorized into a five-level ordinal scale: normal (0–33); mild (33.1–43); moderate (43.1–60); severe (60.1–79) and very severe (≥ 80) according to the Depression, Anxiety and Stress Scale (DASS) [18]. Overall occupational stress was re-categorized into two levels: low stress and excessive stress using the median as a cut-off. Depression was also re-categorized into two levels: no depression (normal:0–15) and depressed (mild:15.1–33; moderate:33.1–52; major:52.1–70; and severe depression:≥ 70.1) based on initial categorization adopted from a previous paper [19]. The Skewness/Kurtosis test for normality was conducted to assess the distributions of each composite variable before categorization. The Chi-square test of independence and Kruskal Wallis test were employed to assess the significance of associations with subsequent sensitivity analysis using Multiple logistic, Poisson and Probit regression models. To estimate sample average treatment effect (occupational stress) on the treated (respondents who reported excessive occupational stress), this study employed Coarsened Exact Matching (CEM) to improve on the estimation of the causal effect of occupational stress on depression and insomnia by reducing the imbalance in covariates between treated and control (respondents who did not report excessive occupational stress) groups. The advantages of using CEM instead of other matching procedures have been well documented [20–22]. The *L*_1_ statistic that measures overall covariate imbalance between workers with excessive stress and those with low stress before and after matching were estimated. The results from the Chi-square test of independence showed an only statistically significant relationship between occupational stress and age of the workers, responsibility for dependents as well as work experience hence the CEM procedure was restricted to these three variables and marital status which had a *p*-value close to the cut-off for statistical significance. However, to improve the precision of our impact estimate and to adjust for the remaining imbalance with other covariates, a Poisson regression model with a robust standard error was used to adjust for these other covariates.

## Results

A total of 235 respondents provided complete data in their self-administered questionnaires (96% response rate) and their demographic characteristics are presented in Table 1. The mean age for the sample was 30.84 ± 6.9 years with 53.6% of respondents within the age range of 20–29 years. More males (52.8%) than females participated in this study just as there were more singles (61.3%) than married. Respondents who reported having at least one dependent at home (40.9%) were fewer than those who had no dependents. For job characteristics, more respondents (59.6%) were permanent employees and 51.9% worked overtime (more than 40 h per week) for an average of 5.24 ± 7.1 h per week. Employees who had worked for a maximum of 5 years were 86.8%.

**Table 1:** Distribution of occupational stress by background characteristics

Characteristics	Occupational stress levels n (%)		χ^**2**^ value, ***p***	Total n (%)
	Low Stress (158)	Excessive Stress (77)		
***Age groups***			7.26, **0.027**	
20–29 years	76 (48.10)	50 (64.94)		126 (53.62)
30–39 years	53 (33.54)	21 (27.27)		74 (31.49)
40–49 years	29 (18.35)	6 (7.79)		35 (14.89)
Mean age ± SD				30.84 ± 6.9
***Sex***			1.48, 0.224	
Males	79 (50.00)	45 (58.44)		124 (52.77)
Females	79 (50.00)	32 (41.56)		111 (47.23)
***Marital status***			3.78, 0.052	
Married	68 (43.04)	23 (29.87)		144 (61.28)
Single	90 (56.96)	54 (70.13)		91 (38.72)
***Dependents***			5.72, **0.017**	
No	85 (53.8)	54 (70.13)		139 (59.15)
Yes	73 (46.2)	23 (29.87)		96 (40.85)
***Employment***			2.77, 0.096	
Permanent	100 (63.29)	40 (51.95)		140 (59.57)
Contract	58 (36.71)	37 (48.05)		95 (40.43)
***Job ranking***			3.42, 0.064	
Other	129 (81.65)	70 (90.91)		199 (84.68)
Superior	29 (18.35)	7 (9.09)		36 (15.32)
***Hours per week***			0.68, 0.408	
Regular (40 h per week)	73 (46.2)	40 (51.95)		113 (48.09)
Overtime	85 (53.8)	37 (48.05)		122 (51.91)
Mean hours overtime±SD				5.24 ± 7.1
^**a**^***Work experience: median (LQ, UQ)***	2 (0.75,4.5)	1 (0.67,2)	**0.0013**	1.17 (0.75,4)

*SD* Standard Deviation; ^a^ The duration of service (years) in the current role; *LQ* Lower Quartile, *UQ* Upper Quartile; *p* values in bold are statistically significant

All four psychosocial stressors: workload (97.9%), responsibility for others (50.6%), role ambiguity (34.5%) and role conflict (17.0%) were reported by respondents. The prevalence of excessive occupational stress at this telco was 32.8% (95% CI = 26.7–38.8). The distribution of excessive occupational stress by background characteristics among respondents is displayed in Table 1. Most employees who reported excessive stress from their jobs were in the 20–29 years category (64.9%) and more males (58.4%) than females also reported the same. Occupational stress was higher among singles (70.1%), respondents without dependents (70.1%), and respondents on permanent employment (52.0%). Fifty percent of respondents who reported excessive occupational stress had worked for at least 1 year in their current role. The Chi-square test of independence revealed that occupational stress was only significantly associated with age, responsibility for dependents at home and work experience.

More than three-quarters (84%; 95%CI = 78.51–88.03) of the respondents reported depression in the past week. Only 6% (95% CI = 3.24–9.37) of respondents reported being diagnosed with insomnia in the past 1 year. Table 2 shows that employment status, age, marital status and responsibility for dependents were the background characteristics significantly associated with depression whereas only marital status and responsibility for dependents were significantly associated with insomnia. Occupational stress was also significantly associated with insomnia but not depression. After controlling for the effect of other variables, sex predicted the highest risk of depression. Table 3 displays the effect of CEM on the relative risk (RR) predicted by each variable. There was a 20% [ARR; 95% CI = 1.20 (1.06–1.36)] increased risk of reporting depression among females compared to males after conducting Poisson regression with CEM. However, of all variables, occupational stress was the least predictor of depression. The estimated risk of reporting depression among employees who reported being exposed to excessive stress from work was 5% [ARR; 95% CI = 1.05 (0.94–1.17)] after adjusting for other background characteristics. Moreover, this risk reduced to 3% [ARR; 95% CI = 1.03(0.91–1.17)] after Poisson regression with CEM. All background characteristics in exception of occupational stress and job ranking predicted higher RR for depression after conducting this procedure. Generally, the odds ratios (ORs) were far higher than the RRs.

**Table 2:** Bivariate analysis of factors associated with depression and insomnia using the Chi-square test of independence

Characteristics	Depression n(%)		χ^**2**^, ***p***	Insomnia n (%)		χ^**2**^, ***p***
	No (38)	Yes (197)		No (221)	Yes (13)	
***Overall occupational stress***			0.86, 0.355			5.11, **0.024**
Low	28 (73.68)	130 (65.99)		152 (68.78)	5 (38.46)	
Excessive	10 (26.32)	67 (34.01)		69 (31.22)	8 (61.54)	
***Age groups***			9.71, **0.008**			0.064*
20–29 years	13 (34.21)	113 (57.36)		29 (25,36)*	26 (25,27)*	
30–39 years	20 (52.63)	54 (27.41)				
40–49 years	5 (13.16)	30 (15.23)				
***Sex***			3.08, 0.079			0.23, 0.634
Males	25 (65.79)	99 (50.25)		117 (52.94)	6 (46.15)	
Females	13 (34.21)	98 (49.75)		104 (47.06)	7 (53.85)	
***Marital status***			11.4, **0.001**			8.60, **0.003**
Married	24 (63.16)	67 (34.01)		90 (40.72)	0	
Single	14 (36.84)	130 (65.99)		131 (59.28)	13 (100)	
***Dependents***			3.90, **0.048**			6.18, **0.013**
No	17 (44.74)	122 (61.93)		127 (57.47)	12 (92.31)	
Yes	21 (55.26)	75 (38.07)		94 (42.53)	1 (7.69)	
***Employment***			7.06, **0.008**			0.03, 0.872
Permanent	30 (78.95)	110 (55.84)		131 (59.28)	8 (61.54)	
Contract	8 (21.05)	87 (44.16)		90 (40.72)	5 (38.46)	
***Job ranking***			0.01, 0.930			0.63, 0.429
Other	32 (84.21)	167 (84.77)		186 (84.16)	12 (92.31)	
Superior	6 (15.79)	30 (15.23)		35 (15.84)	1 (7.69)	
***Hours per week***			0.65, 0.420			1.61, 0.204
Regular	16 (42.11)	97 (49.24)		108 (48.87)	4 (30.77)	
Overtime	22 (57.89)	100 (50.76)		113 (51.13)	9 (69.23)	
***Work experience: median (LQ, UQ)***	2 (1,4)	1.2 (0.67,3.5)	0.120	1.3 (0.75,4.0)	1 (0.67,1.25)	0.100
***Alcohol intake***			1.79, 0.181			2.50, 0.114
No	34 (91.89)	164 (83.25)		189 (85.52)	9 (69.23)	
Yes	3 (8.11)	33 (16.75)		32 (14.48)	4 (30.77)	
***Smoking status***			2.80, 0.094			0.07, 0.789
No	37 (100)	183 (92.89)		208 (94.12)	12 (92.31)	
Yes	0	14 (7.11)		13 (5.88)	1 (7.69)	

* The Kruskal Wallis test was conducted to assess if insomnia significantly differed between age groups; *p* values in bold are statistically significant

**Table 3:** Estimating the impact of occupational stress on depression: Modified Poisson regression with CEM

Variables	Depression			
	Poisson regression with CEM	Sensitivity Analysis		
		Ordinary Poisson regression analysis	Logistic regression analysis	Probit regression analysis
	RR [95% CI]	RR [95% CI]	OR [95% CI]	β [95% CI]
***Occupational stress***				
Low	Ref	Ref	Ref	Ref
Excessive	1.03 [0.91–1.17]	1.05 [0.94–1.17]	1.43 [0.61–3.39]	0.23[− 0.25–0.71]
***Age groups***				
18–29 years	^a^	Ref	Ref	Ref
30–49 years		0.95 [0.81–1.12]	0.83 [0.27–2.52]	− 0.07[− 0.69–0.55]
***Sex***				
Males	Ref	Ref	Ref	Ref
Females	1.20 [1.06–1.36]	1.11 [0.99–1.24]	2.11 [0.96–4.67]	0.42 [0.02–0.86]
***Marital status***				
Married	^a^	Ref	Ref	Ref
Single		1.28 [1.03–1.59]*	4.04 [1.22–13.33]*	0.78 [0.10–1.46]*
***Dependents***				
No	^a^	Ref	Ref	Ref
Yes		1.10 [0.91–1.34]	1.61 [0.53–4.92]	0.27[−0.36–0.91]
***Employment***				
Permanent	Ref	Ref	Ref	Ref
Contract	1.14 [1.01–1.29]	1.11 [0.99–1.26]	2.43 [0.88–6.65]	0.50[− 0.04–1.05]
***Job ranking***				
Other	Ref	Ref	Ref	Ref
Superior	1.08 [0.94–1.23]	1.13 [0.95–1.34]	1.99 [0.68–5.82]	0.39[− 0.21–0.99]
***Hours per week***				
Regular	Ref	Ref	Ref	Ref
Overtime	1.06 [0.94–1.21]	0.96 [0.86–1.07]	0.74 [0.34–1.60]	−0.12[− 0.54–0.31]
***Work experience***	^a^	1.02 [1.00–1.04]	1.13 [0.96–1.33]	0.07[−0.02–0.16]
***Alcohol intake***				
No	Ref	Ref	Ref	Ref
Yes	1.13 [0.97–1.33]	1.11 [0.98–1.25]	2.23 [0.59–8.37]	0.7[−0.24–1.17]

*P*-value notation: ***p < 0.001; **p < 0.01, *p < 0.05

^a^: these variables were excluded from the model due to CEM

The RRs from ordinary Poisson regression analysis were lower compared to ORs for insomnia predicted by most of the variables. Nonetheless, the two measures of association were similar for job ranking, type of employment, work experience, smoking status and responsibility for dependents. After conducting Poisson regression with CEM analysis, similar to what was observed for depression, the RR predicted by occupational stress and employment type reduced whereas it increased for all other variables. Controlling for background characteristics, employees who reported excessive occupational stress had 2.58 times the risk of reporting insomnia [ARR; 95% CI = 2.58 (0.83–8.00)] compared to those who reported low stress from their jobs and this risk reduced to 2.46 [ARR; 95% CI = 2.46 (0.77–7.87)] after employing Poisson regression with CEM. Alcohol intake posed the highest risk of reporting insomnia compared to other background characteristics whereas employment posed the least risk (Table 4).

**Table 4:** Estimating the impact of occupational stress on Insomnia: Modified Poisson regression with CEM

Variables	Insomnia			
	Poisson regression with CEM	Sensitivity Analysis		
		Ordinary Poisson regression analysis	Logistic regression analysis	Probit regression analysis
	RR [95% CI]	RR [95% CI]	OR [95% CI]	β [95% CI]
***Occupational stress***				
Low	Ref	Ref	Ref	Ref
Excessive	2.46 [0.77–7.87]	2.58 [0.83–8.00]	3.00 [0.88–10.28]	0.52[− 0.10–1.14]
***Age groups***				
18–29 years	^a^	Ref	Ref	Ref
30–49 years		0.61 [0.06–5.90]	0.57 [0.08–3.84]	−0.28[−1.18–0.61]
***Sex***				
Males	Ref	Ref	Ref	Ref
Females	2.05 [0.64–6.62]**	1.58 [0.53–4.76]	1.72 [0.47–6.30]	0.31[−0.35–0.96]
***Dependents***				
No	^a^	Ref	Ref	Ref
Yes		0.25 [0.02–3.45]	0.22 [0.02–2.21]	−0.77[−1.81–0.28]
***Employment***				
Permanent	Ref	Ref	Ref	Ref
Contract	0.70 [0.19–2.49]*	0.36 [0.11–1.18]	0.30 [0.08–1.16]	−0.59[−1.27–0.10]
***Job ranking***				
Other	Ref	Ref	Ref	Ref
Superior	0.53 [0.08–3.44]	0.57 [0.07–4.66]	0.52 [0.05–5.15]	−0.39[−1.55–0.77]
***Hours per week***				
Regular	Ref	Ref	Ref	Ref
Overtime	2.29 [0.70–7.52]	2.07 [0.65–6.62]	2.32 [0.62–8.73]	0.43[−0.23–1.09]
***Work experience***	^a^	0.72 [0.51–1.02]	0.69 [0.39–1.24]	−0.17[− 0.47–0.13]
***Alcohol intake***				
No	Ref	Ref	Ref	Ref
Yes	3.15 [0.78–12.73]	1.77 [0.41–7.74]	1.95 [0.44–8.69]	0.38[−0.37–1.13
***Smoking status***				
No	Ref	Ref	Ref	Ref
Yes	0.27 [0.03–2.91]	0.54 [0.04–6.63]	0.49 [0.05–5.41]	−0.27[−1.40–0.86]

*P*-value notation: ***p < 0.001; **p < 0.01, *p < 0.05

^a^: these variables were excluded from the model due to CEM

## Discussion

The results of the study have revealed that almost a third (32.8%) of employees at this telco were excessively stressed from work and more than three-quarters reported depression in the past week, whereas only a few reported being diagnosed with insomnia in the past year. Age, having dependents at home and work experience were the only respondent characteristics that were significantly associated with exposure to occupational stress. This study also found that occupational stress was only a predictor for reported insomnia but not depression among respondents. There was a higher risk of insomnia among those exposed to excessive stress from work compared to those who reported lower exposure. In Africa, a similar prevalence (28.2%) has been recorded among university staff in Ethiopia [23], and higher among Tanzanians (46.5%) [24]. In Asia, prevalence rates varied from 65.8% among Indian call centre workers [25] to 21.7% among university staff in Malaysia [26]. Physicians (51.4%) and nurses (51.2%) who worked in the Intensive Care Unit and anaesthesiology unit at hospitals in Poland [27] were also reported to have work-related stress. However, there is insufficient evidence of the prevalence of occupational stress among telco employees in Africa.

In the current study, workload was the most reported of the four psychosocial stressors assessed whereas role conflict was the least source of stress. This finding is consistent with the observations of other researchers [12, 28] who also reported workload as a major stressor in telcos and the hotel industry respectively. Occupational stress was significantly associated with age, having dependents at home and work experience of the employee. Younger employees may have spent a shorter length of time in their roles and may yet be acquiring the needed skills for effective delivery at work as compared to their older counterparts and therefore may be more stressed [29]. Similar reasoning is drawn from Sharma and Devi’s [2] finding that a longer duration spent in a job role was inversely related to the job stress reported. Also, employees with dependents may have more stringent allocations for time due to their extra responsibilities at home. Consequently, they may function more effectively under stress compared to those without dependents [30].

Among the respondents of this study, excessive occupational stress was not found to be significantly associated with depression. Tsai, Chi, and Wang [30] reported a similar finding from a longitudinal study conducted on a sample of the older working population in Taiwan. They indicated that their study population comprised of employees nearing retirement (≥50 years) who were more likely to be experiencing diminishing job stress as earlier posited [31]. However, they attributed the highest impact on depression to perceived-health stress which was not assessed by this study. Other findings based on cross-sectional [32, 33] and longitudinal data [34] reported, contrary to this study, a strong positive association between work-related stress and depression. Yoshizawa et al. [32] specifically indicated social support, job control, and quantitative workload as psychosocial stressors significantly influencing depression among some psychiatric nurses in Japan. Oenning et al. [33] and Romswinkel et al. [34] assessed job stress in general and among community-dwelling workers as opposed to employees from a specific industry which is the case for this current study. In a lower-middle-income country like Egypt, a positive correlation between work stress and depression was also reported [35]. Similar to this study, these researchers assessed both depression and occupational stress using self-reports except for Oenning et al. [33] who used a diagnostic tool to measure depressive disorder. The robust risk estimation from this study may account for the lack of association between occupational stress and depression. However, the effect of residual confounding on this association from risk factors like income level, educational level, life events, personality traits, earlier psychiatric morbidity and family history [36] which were not measured and controlled for in this study cannot be overlooked. Moreover, other stress parameters not measured in the current study such as effort, reward, overcommitment and support were found to influence the level of depression among workers exposed to high levels of occupational stress whereas the workload (demand) was less important than the other variables [37].

Occupational stress has been reported to facilitate the development of sleep problems [7]. Elger and Sekera [38] indicated that stressful events predicted insomnia, the most prevalent sleep disorder [39]. Also, a recent systematic review involving fifteen (15) countries found a direct relationship between occupational exposure to violence as a stress factor and sleep problems [40]. The current study identified occupational stress as an important risk factor of insomnia. Almost all employees reported quantitative workload as a stressor. The aftermath of this exposure to stress could have been after-work fatigue which influences the experience of insomnia. Also, high levels of work rumination may advance worry [9]. The daily psychological strain as a consequence of worrying about workload could have contributed to this occupational stress-related insomnia observed in this study. This is informative to employers especially in telcos of the possible effect of workload strain on workers’ psychological health. It has been proven that reducing weekly work hours of employees by a quarter could significantly reduce workload and increase the time they spend on recovery activities on weekdays [41], improve sleep quality [42] and consequently reduce occupational stress [43]. Our study indicated a higher risk of insomnia among those who worked overtime compared to those who worked regular hours. Hence, employers in Ghanaian telcos could curb occupational stress and its increased risk of insomnia by putting measures in place to ensure employees spend minimal time working beyond regular hours.

### Implications for future research

There is a paucity in literature regarding the impact of occupational stress on depression and insomnia in telcos, especially in Africa. To this end, this study provides preliminary findings that can form the basis for further work, probably longitudinal studies, to document a clear linkage between occupation-related stress and depression as well as insomnia. This will help report on their peculiar experiences of strain and the two psychiatric conditions. Generally, the odds ratios were far higher than the risk ratios estimated from the Poisson regression with CEM in this study, suggesting an overestimation of the strength of prediction by the variables if only odds ratios were used. This analytical method could be adopted in cross-sectional studies to increase the weight of evidence produced on the predictive power of variables and make them comparable to estimates from longitudinal studies.

### Study limitations

The findings from this study were mainly based on self-reported measures which are highly subjective. Also, the study categorized stress by dividing the population into two parts using the median as cut-off which is a limitation. Moreover, the evidence from this study as is the case for other cross-sectional studies needs supplementation from longitudinal studies. The use of findings from this observational study to estimate relative risk should be interpreted with caution as it may be associated with reverse causality bias although a rigorous statistical procedure was used.

## Conclusion

About a third of employees at the telco studied reported excessive occupational stress whereas depression was reported by over three-quarters of them. Only a few reported being diagnosed with insomnia in the past year. Occupational stress increases the risk of insomnia. However, this study did not find a statistically significant relationship between depression and occupational stress. Findings from this study are informative to employers in the Ghanaian telecommunication sector to conduct routine assessments of the mental health of their employees and explore psychosocial hazards reported by them. This will contribute to effective interventions, including ensuring employees spend minimal time working beyond regular hours, to protect their health holistically.

## Data Availability

The datasets used and analyzed during this study are available from the corresponding author on reasonable request.

## Abbreviations

CEM: Coarsened Exact Matching
CES-D: Center for Epidemiologic Studies Depression Scale
DASS: Depression Anxiety Stress Scales
GJSQ: Generic Job Stress Questionnaire
LMICs: Low-Middle Income Countries
NIOSH: National Institute for Occupational Safety and Health
Telcos: Telecommunication Companies
OR: Odds Ratio
RR: Relative Risk
ARR: Adjusted Relative Risk
